# Liquid Level Measurement Model Outside of Closed Containers Based on Continuous Sound Wave Amplitude

**DOI:** 10.3390/s18082516

**Published:** 2018-08-01

**Authors:** Yanjun Zhang, Bin Zhang, Liang Zhang, Yunchao Li, Xiaolong Gao, Zhaojun Liu

**Affiliations:** 1 Key Laboratory of Instrumentation Science & Dynamic Measurement, Ministry of Education, North University of China, Taiyuan 030051, China; zhangyanjun@nuc.edu.cn (Y.Z.); zhangliang_ty@163.com (L.Z.); yun_chaoli@163.com (Y.L.); Gao_x_l@126.com (X.G.); L1187091247@163.com (Z.L.); 2 Science and Technology on Electronic Test & Measurement Laboratory, North University of China, Taiyuan 030051, China; 3 Software School of North University of China, Taiyuan 030051, China

**Keywords:** ultrasonic, echo energy, liquid level measurement, continuous wave

## Abstract

This research put forward an exogenous liquid level measurement method based on continuous sound wave amplitude. The distribution of round piston transducers in the sound field of a metal solid was analyzed by building 15 Multi-Gaussian Beam superposition models; the calculation method for echo sound pressure was worked out according to the reflection and refraction properties of ultrasonic wave. The continuous wave with three amplitudes was used as the driving source of ultrasonic sensor, and two single-crystal sensors with the same diameter were used as the transmitting terminal and receiving terminal of ultrasonic waves to carry out experiments for four groups of containers of different wall thickness and to compare the characteristics of echo energy of driving sources with three amplitudes above and below the liquid levels with different wall thickness. Two groups of sensors of different diameters were used to measure the liquid levels of experimental models, and the measuring errors of the two groups of sensors were analyzed and compared. The experimental result shows that the measuring error of the model is less than 5 mm, so it is applicable to the level measurement of liquids or liquid mixtures in many sectors.

## 1. Introduction

Height and volume measurements of material in stocking cans and other containers are usually taken in storage of chemicals, food, and petroleum in the production process to make sure of material supply [1,2].

Factors influencing measurement accuracy are to be considered to be the sealing medium of the container, materials of corrosive substance, toxicity, and explosiveness [3,4]. In order to meet requirement of various kinds of influencing factors, liquid level sensors are developed. At present, commercial liquid level measurements include radar, ultrasonic, isotope or radioactive, electronic, thermal, optical meters, and hydraulic meters. Although they achieve successful application, they have to be set into the container connecting with liquid directly, or in need of sound waves and electromagnetic waves [5,6].

Special applicant conditions in petroleum, chemicals, energy, aerospace and other industries call for specialized measurement and equipment of liquid levels. For the conditions of flammables and explosives, ultra-low temperature, high pressure, and other application characteristics of the liquid, the storage should be settled in large, closed containers and a low temperature, high pressure environment away from electrical, magnetic, collision, and other dangerous parts, which put forward higher requirements on level measurement methods and principles.

Given the strengths and weaknesses of these methods, this study presents a detection model for determining the liquid level from the outside of a sealed container that is based on continuous sound wave amplitude, and the proposed method needn’t install sensors or equipment in a container in advance, nor damage the physical structure and integrity of the container [7,8].

This study uses the multivariate Gaussian sound beam model to simulate the circular piston-type transducer in the radiation sound field in the container wall. According to the shape and propagation characteristics of beam propagation, the influence of container walls with different curvatures on the radiation field is analyzed. Based on the difference of ultrasonic impendences between gas and liquid media in a container, the echo sound pressure calculation model is built up with continuous sound wave amplitude [9,10,11,12].

**Figure 1:**
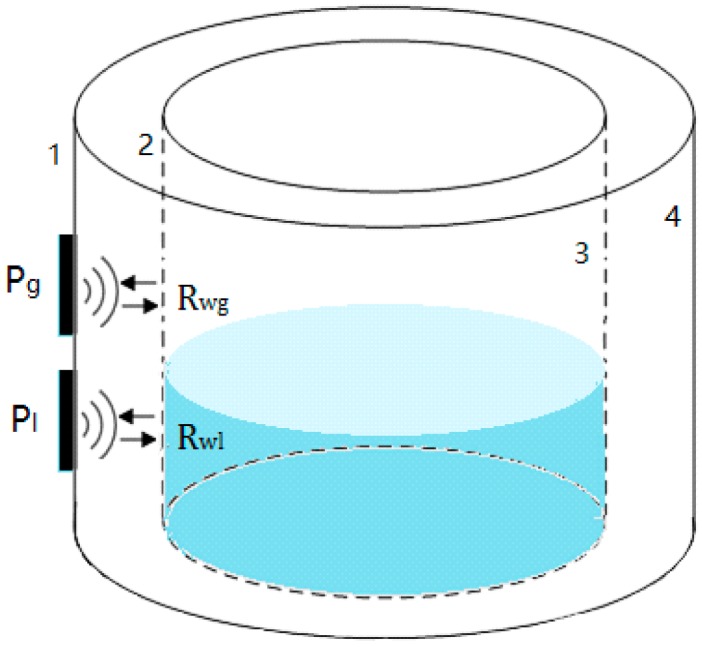
The measurement principle. $P_{g}$ and $P_{l}$ are the sound pressure relating to the echoes reflected by the inner surface of the container.

As shown in Figure 1, $R_{\mathrm{wg}}$ represents the reflection coefficient at the inner surface above the liquid level; $R_{\mathrm{wl}}$ refers to the reflection coefficient below the liquid level. When the sensor is located above and below the liquid level respectively, the reflection coefficients $R_{\mathrm{wg}}$ and $R_{\mathrm{wl}}$ at the inner surface are not equal due to the different impendences, which make the sound pressure relating to received echoes different. Then at a critical point above the liquid level, the echo wave sound pressure keeps constant, and there is a point under the liquid level. Therefore, the changing characteristics of sound pressure in the detection are used to measure the liquid level.

## 2. Theory and Methods

### 2.1. Sound Field of a Round Piston Transducer in a Solid

In the fifteen multivariate models of Gauss beam superposition [13], magnitude of sound pressure at any point (x_2_, y_2_, z_2_) in the solid sample can be expressed as (1)

(1) $$\begin{array}{cc} P\left(x_{2},y_{2},z_{2}\right)= & \frac{\rho_{2}c_{\gamma 2}}{\rho_{1}c_{p1}}\cdot P_{0}\cdot T_{12}^{\gamma ;p}\\ & \cdot \overset{15}{\underset{r=1}{\sum }}\frac{A_{r}}{1+{\mathrm{iz}}_{1}B_{r}/D_{R}}\cdot \frac{\sqrt{Z_{1}^{r}}}{\sqrt{Z_{1}^{r}+z_{2}\left(c_{\gamma 2}/c_{p1}\right)}}\cdot \frac{\sqrt{Z_{2}^{r}}}{\sqrt{Z_{2}^{r}+z_{2}\left(c_{\gamma 2}/c_{p1}\right)}}\\ & \cdot \exp\left[{\mathrm{ik}}_{p1}z_{1}+{\mathrm{ik}}_{\gamma 2}z_{2}+i\frac{k_{p1}}{2}y^{T}{\left[c_{p1}M_{2}^{\gamma }\left(z_{2}\right)\right]}_{r}y\right] \end{array}$$

where $A_{r}$ and $B_{r}$ are superposition coefficient of the multivariate Gauss beam, $T_{12}^{\gamma ;p}$ is the plane wave transmission coefficient. The parameter $D_{R}=k_{p1}a^{2}/2$ is the Rayleigh distance, a is the radius of the transducer, and $k_{p1}$ is the wave numbers for P-waves in medium one. Similarly $k_{\gamma 2}\left(\alpha =p,s\right)$ are wave numbers for P-waves or S-waves in medium two. Z_1_ is the travel length of test sound beam in the first medium; Z_2_ is the length in the second medium. $P_{0}$ is the initial incident sound pressure. $\rho_{1},\;\rho_{2}$ are the medium density, $C_{p1}$ is the wave velocity of the longitudinal wave in the liquid, $C_{\gamma 2}$ is the longitudinal or shear wave velocity in the firmware sample. $M_{2}^{\gamma }$ is a 2 × 2 matrix related to the angle of incidence of the ultrasound beam and the curvature of the interface.

For further discussion, the equation is simplified as

(2) $$\begin{array}{cc} G\left(x_{2},y_{2},z_{2}\right)= & \overset{15}{\underset{r=1}{\sum }}T_{12}^{\gamma ;p}\cdot \frac{A_{r}}{1+{\mathrm{iz}}_{1}B_{r}/D_{R}}\cdot \frac{\sqrt{Z_{1}^{r}}}{\sqrt{Z_{1}^{r}+z_{2}\left(c_{\gamma 2}/c_{p1}\right)}}\\ & \cdot \frac{\sqrt{Z_{2}^{r}}}{\sqrt{Z_{2}^{r}+z_{2}\left(c_{\gamma 2}/c_{p1}\right)}}\\ & \cdot \exp\left[{\mathrm{ik}}_{p1}z_{1}+{\mathrm{ik}}_{\gamma 2}z_{2}+i\frac{k_{p1}}{2}y^{T}{\left[c_{p1}M_{2}^{\gamma }\left(z_{2}\right)\right]}_{r}y\right] \end{array}$$

**Figure 2:**
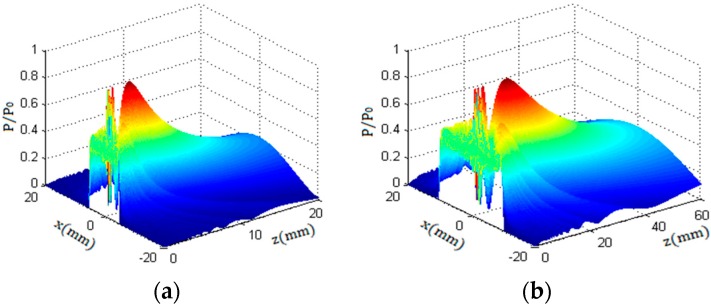
The Sound pressure distribution of a round piston transducer with a radius of a = 5 mm and 10 mm in a solid medium. (**a**) a = 5 mm; (**b**) a = 10 mm.

Figure 2 shows a three-dimensional view of the sound field superimposed by 15 Gauss sound beams. In Figure 2, a and b indicate the sound field distribution of a circular piston probe in metallic aluminum. The compressional wave speed was 6300 m/s, the shear wave speed was 3100 m/s, and the ultrasonic impedance was 17 × 105 gm/cm^2^·s.

From analysis of the sound field distribution theory and Figure 2 of the 3D view, we know that the sound field of the circular piston sensor consists of two parts, the near field and the far field; and there are many maximum and minimum sound pressures in the near field. In the far field, the sound pressure decreases gradually with the increase in propagation distance. Since the sound field of the circular transducer is symmetrical along its axis, the sound pressure distribution characteristics of any cross section of the beam can be obtained along the propagation direction of the beam [14].

Then the beam at near-field keeps cylindrical propagation with little divergence; the beam at far-field spreads in a diffuse manner with a certain diffusion angle. The length of the near field N and the diffusion angle α are given by $N=a^{2}/\lambda_{c}$ and $\alpha =\arcsin\left(1.22\lambda_{c}/2a\right)$, respectively, where $\lambda_{c}$ is the wavelength of ultrasonic waves in a metal wall, a is the radius of the sensor [15].

From the above analysis, it can be inferred that, along the propagating direction of the sound beam, any of the cross sections of the ultrasonic beam are in the circular region. Therefore, when the sound beam arrived at the inner surface of a container after a propagating distance, the projection is a circle section, in which the beam energy is mainly concentrated. We called the projected circle an energy circle, of which the diameter is expressed by d, and the value of d can be calculated by Equation (3):

(3) d=2[a+(L−N)tanβ]  (L>N)

### 2.2. Calculating Echo Sound Pressure

Since the Gaussian sound beam is perpendicular to the flat interface, its reflection and transmission characteristics follow the propagation of plane waves [16,17]. Therefore, the reflection coefficient is calculated as follows:

(4) $$R_{w}=\frac{\rho_{2}c_{2}\cos\theta_{i}-\rho_{1}c_{1}\cos\theta_{r}}{\rho_{2}c_{2}\cos\theta_{i}+\rho_{1}c_{1}\cos\theta_{r}}$$

where $c_{1},\;c_{2}$ are the speed of sound in the medium, $\theta_{i}$ is the incidence angle, $\theta_{r}$ is the reflection angle.

**Figure 3:**
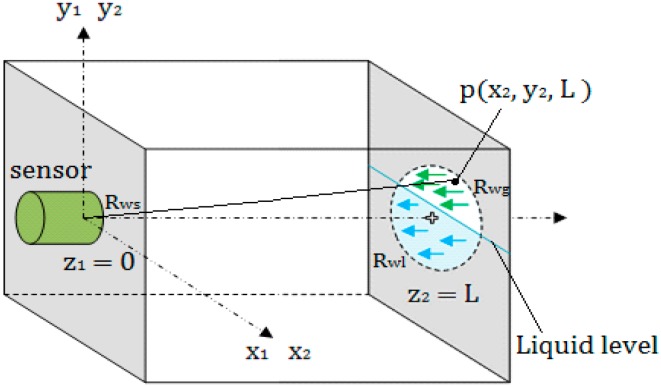
Calculation of echo energy.

As shown in Figure 3, it is supposed that the wall thickness of the container is L; the radius of the sensor is a; the coordinate system is established by taking the center O of the circular transducer as the origin of coordinates. According to the multivariate Gaussian beam model, let $z_{1}=0$, $z_{2}=L$, the ultrasonic pressure $P_{L}$ emitted by the circular sensor at any point $p\left(x_{2},y_{2},z_{2}\right)$ on the inner wall of the container with the wall thickness L can be expressed as

(5) $$P_{L}\left(x_{2},y_{2},L\right)=\frac{\rho_{2}c_{\gamma 2}}{\rho_{1}c_{p1}}\cdot P_{0}\cdot G\left(x_{2},y_{2},L\right)$$

According to the above analysis of the energy circle, the sound pressure in the circular beam section of diameter d is integrated; assuming that the total area of the energy circle is represented by and the average sound pressure in the energy circle is obtained as follows:

(6) $$\bar{P}=\frac{\int_{S}P_{L}\left(x_{2},y_{2},L\right)\mathrm{dA}}{A}$$

According to the basic knowledge of acoustics, the ultrasonic wave is refracted and reflected at the interface with discontinuous impedance, which follows the refraction and reflection principle of plane sound waves. To assume the average reflection echo sound pressure at the inner wall of the container as $\bar{p_{r}}$, then

(7) $$\bar{p_{r}}=\bar{P}\cdot R_{w}$$

In actual detection, when the sensor is moved up along the outer surface of the wall and the top of the energy circle exceeds the liquid level, the excess height is represented by Δd (0≤Δd≤d), the area above the liquid level is expressed $A_{e}$, let $r_{s}=A_{e}/A$.

As 0≤Δd≤d, the “energy circle” is divided into two parts by the liquid level, the acoustic impedances are no longer equal at two parts of the energy circle, which will cause the acoustic boundary conditions of two parts also different. Therefore, it is calculated in two parts, and represents reflection coefficients at two parts of the energy circle; the echo sound pressure received by the sensor should be superimposed by two parts of energy circle. 

We assumed that the reflected echoes in the wall will be decayed to a very small amount after n times which can be negligible relative to the total energy received by receiving sensor. 

Therefore, when the sound beam returns to the outer surface of the wall after n times, the total sound pressure of the sensor is derived by following equation:

(8) $${\Sigma P}_{s}=\bar{P}\cdot A_{s}\cdot \left(r_{s}\cdot \overset{n}{\underset{i=1}{\sum }}R_{\mathrm{wg}}^{i}R_{\mathrm{ws}}^{i-1}e^{-\left(2i-1\right)\alpha L}+(1-r_{s})\cdot \overset{n}{\underset{i=1}{\sum }}R_{\mathrm{wl}}^{i}R_{\mathrm{ws}}^{i-1}e^{-\left(2i-1\right)\alpha L}\right)$$

where, the ultrasonic attenuation factor at the container wall represents the sensor surface reflection coefficient.

## 3. Experimental Results

### 3.1. System Installation and Initial Conditions

**Figure 4:**
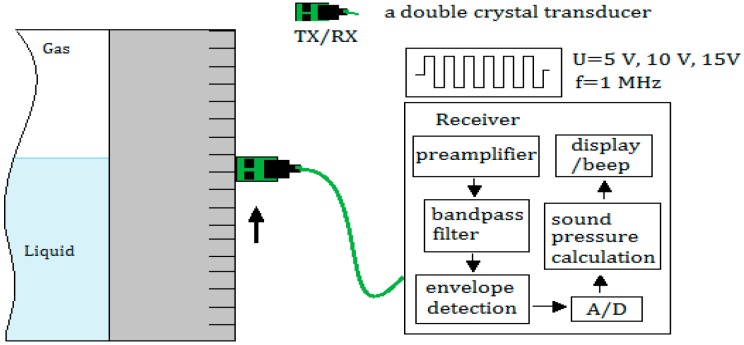
Circuit, metal container, and sensors used in the experiment.

Figure 4 shows the composition of the experimental system.

Four groups of containers of different wall thicknesses at 8 mm, 25 mm, 40 mm, and 50 mm are used for the measurement in the experiment; the substance of the container is aluminum alloy, with liquid as the liquid medium in the container and atmosphere as gas medium. Parameters in Table 1 display the impedance of the metal container $Z_{m}$, the impedance of liquid media in the container $Z_{l}$, the impedance of gas media in the container $Z_{g}$, the reflection coefficient between the inner wall and gas $R_{\mathrm{wg}}$, the reflection coefficient between the inner wall and liquid $R_{\mathrm{wl}}$, and the reflection coefficient between the outer wall and probe $R_{\mathrm{ws}}$.

The driving source is designed as continuous wave amplitude excitation, that is, the amplitude of continuous wave is sequentially taken in three cycles of 5 V, 10 V, and 15 V to drive the probe to emit ultrasonic waves at different amplitude. Due to use of continuous wave excitation, the ultrasonic transducer is used with a dual-crystal probe, one for emission and one for receiving, and both of which have a diameter of 10 mm and a focal length of 50 mm.

**Figure 5:**
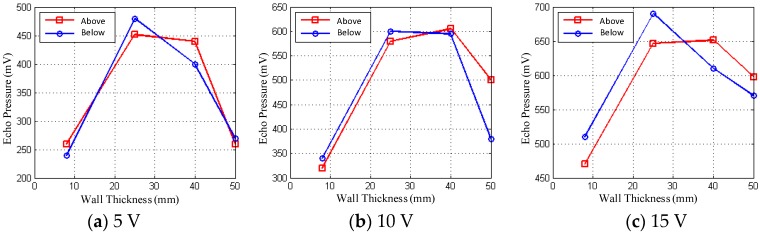
Average echo pressures at various wall thicknesses of the Excitation voltage 5 V, 10 V, 15 V.

As shown in Figure 5, the difference between the two critical values of the echo energy above and below the liquid level shows different characteristics in the given test environment for different values of the amplitude excitation signal. In Table 2, when the three amplitude voltage signals are activated in all areas of the wall thickness of the container, there is always a group of incentive voltage values matched with thick wall, and the liquid level below the echo energy of the two critical values of the difference are of higher resolution, a clear distinction, which provides a basis to judge the liquid level position determination.

### 3.2. Calculation of Echo Sound Pressure

**Figure 6:**
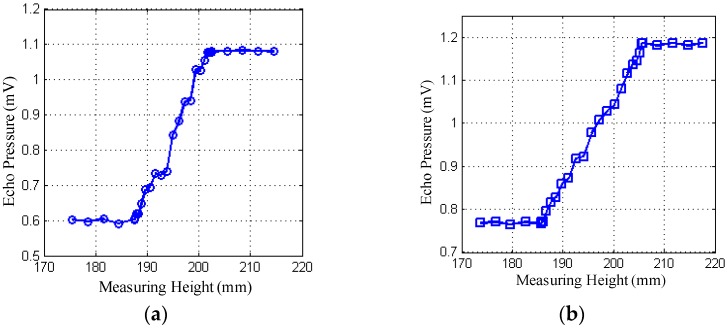

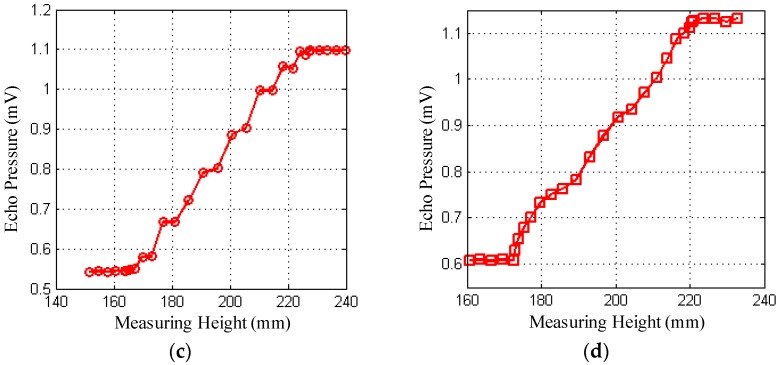
Variation of echo pressure at various wall thicknesses. (**a**) L = 8 mm, 2a = 15 mm; (**b**) L = 8 mm, 2a = 20 mm; (**c**) L = 50 mm, 2a = 15 mm; (**d**) L = 50 mm, 2a = 20 mm.

Figure 6 shows the actual measurements of probes at different sizes for different wall thicknesses, with the abscissa representing the height measured by the probe along the outer wall of the container and the ordinate representing the amplitude of the echo pressure received by the receiving probe. The ultrasonic echo is received by the receiving circuit. The gain of the echo signal is amplified, filtered, detected for data processing, and converted to the corresponding voltage amplitude display output. In the figure, excitation voltage is set at 15 V for an example, at the wall thickness of L = 8 mm and L = 50 mm.

### 3.3. Level Measurement Results

**Figure 7:**
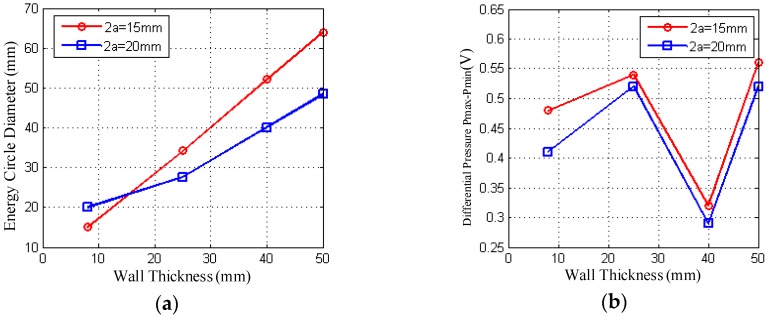

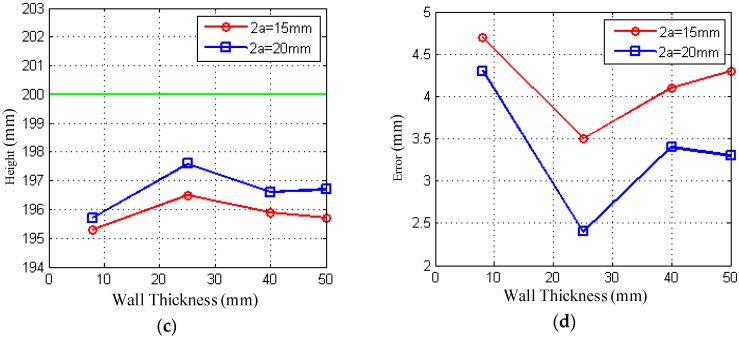
Experimental result. (**a**) Relationship of energy circle diameter and container wall thickness; (**b**) Sound pressure difference between two critical positions at different wall thicknesses; (**c**) Liquid level value at different wall thickness; (**d**) Error at different wall thickness.

Figure 7 shows the true result of liquid level test using the theory model in Section 3. Liquid level in the container is of water, with the actual liquid level at 200 mm. In the experiment, transducers of two different sizes 15 mm and 20 mm in diameter were used to measure the liquid level under the condition of wall thickness L of 8 mm, 25 mm, 40 mm, and 50 mm respectively. ${\bar{P}}_{\max}$ and ${\bar{P}}_{\min}$ in the table (Table 3) are the echo sound pressure values measured at the upper and lower critical positions respectively, $h_{l}$ is the actually liquid level value, ${\bar{h}}_{\max}$ and ${\bar{h}}_{\min}$ are the heights of the upper and lower critical positions of the liquid level respectively. ${\bar{h}}_{m}$ is the actual measurement result, and $\left|\bar{\Delta E}\right|$ is the average error of the measurements. All measurements in the table are the average of three experiments.

Figure 7a shows the corresponding relationship between the diameter d of the energy circle and the wall thickness L of the beam generated by probes of two different diameters for different wall thicknesses. In the figure, the transducer of 15 mm diameter emits ultrasonic beam divergence faster with the propagation distance increase. At the same wall thickness, the diameter d of the energy circle increases linearly, which indicates that the beam spreads quickly and the energy of the sound beam in the unit area of the corresponding energy circle also decreases, that is, the average sound pressure of the beam decreases which will affect the detection resolution. The beam divergence generated by a transducer with a diameter of 20 mm is relatively flat.

Figure 7b shows the correspondence between the echo sound pressure received by two different diameters of the probe at two critical positions and wall thickness L under different wall thicknesses. Two different diameters of the probe in different wall thicknesses showed the same changes. At 40 mm wall thickness, the pressure difference becomes the minimum. At wall thickness of 25 mm and 50 mm, the pressure difference becomes the larger. This is useful for measuring the level and for improving the resolution of the measurement. At wall thickness of 8 mm, the pressure drops at upper and lower critical positions are also more pronounced due to use of delay blocks between the probe and the container wall.

As can also be seen from Figure 7b, the difference between the echo pressures at the two critical positions is generally higher for the different wall thicknesses than for the larger diameter probe when a small diameter probe is used. As the diameter of the transducer increases, the ultrasound beam will become more focused and the divergence angle becomes smaller, while the length of the near field will become longer, the spacing between the two key positions will also be smaller, and the sensitivity of the transducer will change higher, but the resolution will be lower. Conversely, as the transducer diameter decreases, the near-field length decreases and the diffusion angle increases. The ultrasound beam will become more divergent and the spacing between the two key positions will also increase. The sensitivity of the transducer will be lower, while the resolution will be higher.

Figure 7c is a comparison of the test results of two probes with the actual level value. It can be seen from the figure that the two diameters of the probe in the side of the test results are lower than the actual level. The system error is caused by the system’s measurement model. Follow-up correction methods are supposed to be proposed after the error analysis to modify the error value. In addition, because both probes are of a near-field length of more than 8 mm, the delay block is used as a secondary measurement to improve detection accuracy. However, it can be seen from the test results that the measured values at wall thickness L = 8 mm still deviate greatly. From the overall measurement point of view, the result of using a larger diameter probe to measure than using a smaller diameter probe measure is relatively closer to the true level. 

Figure 7d shows the error of detecting the liquid level under four different wall thicknesses by using two kinds of probes respectively. As can be seen from the figure, the measurement error of the two probes reaches about 4 mm~5 mm when the wall thickness L = 8 mm. The measurement error is reduced when wall thickness L ≥ 25 mm. Two different sizes of the probe’s error values have reached minimum when wall thickness L = 25 mm. However, it can be seen from the overall situation that the measurement error of a larger diameter probe is slightly lower than that of a small diameter probe. The measurement error of the two probes generally remains at about 3~5 mm.

## 4. Discussion

In the ultrasonic liquid level meter measurement of this design, the upper and lower critical positions are to be determined first by the measurement of the changing characteristics of the reflection sound wave in the vicinity of the liquid level. In the measurement model, the impedance of liquid medium and atmosphere medium is the major factor influencing measurement precision. The liquid medium of greater impedance, in which reflection sound pressure varies greatly obviously, is easy to determine the upper and lower critical positions and the liquid level; in the liquid medium of smaller resistance, in which reflection sound pressure does not vary obviously, it is difficult to fix the upper and lower critical positions and the liquid level. Therefore, the liquid medium of greater impedance is easier to be determined with higher precision, or vice versa. 

The experimental result shows that, by probes of two different diameter sizes, the regularities of the reflection sound amplitude are similar. When the small diameter probe is used for varied wall thicknesses, the differences of reflection sound are larger than the big diameter probe generally; whereas, the measurement result for liquid shows that the precision of a large diameter probe is better than the small one due to different physical properties of different diameter probes in special test environments to balance the test sensitivity and resolution of the probe in a process. Therefore, in this paper on ultrasonic liquid position measurement, selection of probe diameters depends on wall thickness and other measurements of physical properties to better balance its resolution and sensitivity.

## 5. Conclusions

In this study, under the static measurement condition, the measurement accuracy of the model is less than ±5 mm for many common liquids or mixed liquids in industry; for metal containers, the test thickness reaches up to 2~50 mm. Therefore, the proposed method is effective for liquid level measurement outside a sealed container. 

## Figures and Tables

**Table 1:** Experimental parameters and initial values.

$Z_{m}\left(gm/cm^{2}\cdot s\right)$	$Z_{l}\left(gm/cm^{2}\cdot s\right)$	$Z_{g}\left(gm/cm^{2}\cdot s\right)$	$R_{wg}$	$R_{wl}$	$R_{ws}$
$17\times 10^{5}$	$1.48\times 10^{5}$	$0.0004\times 10^{5}$	0.999969231	0.892285298	0.892285298

**Table 2:** Distinguishing characteristics of variable amplitude excitation under different wall thicknesses.

Distinguishing Characteristics	8 mm	25 mm	40 mm	50 mm
5 V	Average	Obvious	Obvious	Not obvious
10 V	Not obvious	Obvious	Obvious	Average
15 V	Not obvious	Obvious	Not obvious	Obvious

**Table 3:** Result of liquid level test.

2a (mm)	L (mm)	N (mm)	d (mm)	$h_{l}\;(\mathrm{mm})$	${\bar{P}}_{min}\;(V)$	${\bar{P}}_{max}\;(V)$	${\bar{h}}_{min}\;(\mathrm{mm})$	${\bar{h}}_{max}\;(\mathrm{mm})$	${\bar{h}}_{m}\;(\mathrm{mm})$	$\left|\bar{\Delta E}\right|\;(\mathrm{mm})$
15	8	8.93	15.0	200.0	0.6	1.08	186.4	204.2	195.3	4.7
15	25	8.93	34.2	200.0	0.88	1.42	178.9	214.1	196.5	3.5
15	40	8.93	52.1	200.0	0.81	1.13	168.7	223.1	195.9	4.1
15	50	8.93	64.0	200.0	0.54	1.1	163.9	227.5	195.7	4.3
20	8	15.87	20.0	200.0	0.77	1.18	185.7	205.7	195.7	4.3
20	25	15.87	27.59	200.0	0.93	1.45	183.3	211.9	197.6	2.4
20	40	15.87	40.08	200.0	0.92	1.21	176.4	216.8	196.6	3.4
20	50	15.87	48.41	200.0	0.61	1.13	172.7	220.7	196.7	3.3
